# Volatile communication in Actinobacteria: a language for secondary metabolism regulation

**DOI:** 10.1186/s12934-024-02456-4

**Published:** 2024-06-18

**Authors:** Lorena Cuervo, Carmen Méndez, José A. Salas, Carlos Olano, Mónica G. Malmierca

**Affiliations:** 1 Department Functional Biology, University of Oviedo, 33006 Oviedo, Spain; 2 University Institute of Oncology of Asturias (I.U.O.P.A), University of Oviedo, 33006 Oviedo, Spain; 3 Health Research Institute of Asturias (ISPA), 33006 Oviedo, Spain

**Keywords:** Actinobacteria, *Streptomyces* sp., Volatile compounds, Metabolites, Biosynthetic potential, Secondary metabolism, Antibiotic

## Abstract

**Background:**

Volatile compounds are key elements in the interaction and communication between organisms at both interspecific and intraspecific levels. In complex bacterial communities, the emission of these fast-acting chemical messengers allows an exchange of information even at a certain distance that can cause different types of responses in the receiving organisms. The changes in secondary metabolism as a consequence of this interaction arouse great interest in the field of searching for bioactive compounds since they can be used as a tool to activate silenced metabolic pathways. Regarding the great metabolic potential that the Actinobacteria group presents in the production of compounds with attractive properties, we evaluated the reply the emitted volatile compounds can generate in other individuals of the same group.

**Results:**

We recently reported that volatile compounds released by different streptomycete species trigger the modulation of biosynthetic gene clusters in *Streptomyces* spp. which finally leads to the activation/repression of the production of secondary metabolites in the recipient strains. Here we present the application of this rationale in a broader bacterial community to evaluate volatiles as signaling effectors that drive the activation of biosynthesis of bioactive compounds in other members of the Actinobacteria group. Using cocultures of different actinobacteria (where only the volatile compounds reach the recipient strain) we were able to modify the bacterial secondary metabolism that drives overproduction (e.g., granaticins, actiphenol, chromomycins) and/or de novo production (e.g., collismycins, skyllamycins, cosmomycins) of compounds belonging to different chemical species that present important biological activities.

**Conclusions:**

This work shows how the secondary metabolism of different Actinobacteria species can vary significantly when exposed in co-culture to the volatile compounds of other phylum-shared bacteria, these effects being variable depending on strains and culture media. This approach can be applied to the field of new drug discovery to increase the battery of bioactive compounds produced by bacteria that can potentially be used in treatments for humans and animals.

**Supplementary Information:**

The online version contains supplementary material available at 10.1186/s12934-024-02456-4.

## Introduction

The role of volatile compounds (VCs) as communication mediators between organisms is a mechanism that has been widely described among the different kingdoms of living beings [1–3]. The study of these molecules has aroused great interest in the scientific community given their great properties and applications in various fields. Microbial VCs are described as agents that improve plant quality and productivity. Molecules such as acetoin or 2,3-butenediol (produced by *Bacillus* sp.) act as plant growth promoters, which raises interest in horticulture. Another example are pyrazines emitted by *Pseudomonas putida* BP25 that have been reported as immune stimulants inducing plant defenseresponses [4–7]. Volatiles are also used as indicators of quality and safety in areas such as the food industry or acting as reporters of certain diseases in the field of medicine (e.g. *Helicobacter pylori*) [8–10].

The VCs belong to different chemical groups although possess several characteristics in common: high-pressure vapor, low point of ebullition, and low molecular weight. This aforesaid allows easy diffusion on water and air, which determines that are fast–acting chemical messengers [11, 12]. The recognition of these signals has crucial importance at the ecological level since it is a determining fact in the establishment of relationships both at an inter- and intraspecific level. In the microbial world, the exchange of these semiochemicals (information-containing molecules that convey signals for other organisms) acts as a behavioral modulator, determining the survival of certain species or genera in specific environments, as well as their coexistence with other organisms [13, 14]. The effect, therefore, that these compounds can generate on their neighbors can be both negative and positive. Its implications as an inducing agent, among others, have sparked singular interests in the discipline of biomedical research.

Due to the emergence of new diseases and the increase in the appearance of multirresistant pathogens to commonly used treatments, the pharmaceutical industry is claiming new bioactive compounds that can serve as therapeutic alternatives. These inconveniences, along with the continued rediscovery of the same compounds over and over again faced by researchers dedicated to this field, claim a need for different and creative approaches to reach this goal [15–17]. A major source of pharmaceuticals is natural products synthesized by the phylum Actinobacteria, among which, the *Streptomyces* genus is the largest bacterial producer of secondary metabolites with bioactive properties [18, 19]. Other genera such as *Rhodococcus, Micromonospora, Saccharopolyspora, Amycolatopsis, Kitasatospora, Verrucosispora, Pseudonocardia,* and *Salinispora,* among others, also stand out as promising producers [19–21]. Despite the exceptional metabolic potential that this bacterial phylum harbors, it remains untapped because many of the biosynthetic gene clusters (BGCs) involved in the production of secondary metabolites appeared silenced or weakly expressed under standard laboratory conditions [22–26]. This implies the compounds produced by those BGCs remain unknown and therefore their promising properties. Thus, unexploited secondary-metabolism pathways could shelter the production of new active ingredients that could serve as therapeutic alternatives. This is the ground why great efforts have been recently focused on applying different tools to activate these silenced metabolic pathways.

Different genetic tools have been applied to activate silent clusters:, gene overexpression by constitutive active promoters, heterologous expression of global or specific regulators, knockout of repressor genes, etc. Additionally, combinatorial biosynthesis strategy is an excellent option for obtaining new and improved derivatives from other molecules [22, 23, 27–29]. These genetic approaches have also been combined with the search for producers in little-explored environments (deserts, marine sediments, etc.) or in association with other organisms [19, 30, 31]. Microorganisms isolated from these environments may present differential characteristics compared to the traditional soil-isolated actinomycetes that allow their survival in this very particular condition. Additionally, some molecules can act as elicitors, inducing the expression of some clusters, that can be applied during the culture of the producer microorganism. There is a great variety of compounds of different chemical structures that can act as expression inducer agents, among which we highlight the VCs, as they have been presented as an interesting alternative to the use of elicitors in solution more widely used in screening programs for the search for new compounds.

VCs can act as signaling molecules inducing behavioral changes and responses in other neighboring bacteria [6, 32, 33]. As reported on several occasions, volatile compounds produced by microorganisms can exert changes in growth, gene expression, and metabolite production in other bacteria. In our previous work, [34] it was reported how volatile compounds from different *Streptomyces* strains triggered the overproduction of secondary metabolites as well as the production of new ones in other related streptomycetes that shared the same ecological niche. The aim of the present work is to delve into the effect of these volatiles as modulators of microbial not only between bacteria of the same genus but also between bacteria of different genera within the Actinobacteria phylum. Due to *Streptomyces* sp. great variety in metabolite production, this work has focused more on the analysis of the effects on streptomycetes to the detriment of the other Actinobacteria. By co-culturing these bacteria and performingsubsequent chromatographic analysis of the metabolites produced, we have demonstrated the potential of these VOCs as activators of metabolic pathways between different bacterial genera, expanding the usefulness of their application in the field of new drug discovery [19–21, 34].

## Material and methods

### Strains and culture conditions

Eleven *Streptomyces spp.* species used in this work belong to an in-lab Carlos Sialer (CS) collection isolated from the cuticle of leafcutter ants from the tribe *Attini* (*Streptomyces sp.* CS014, CS057, CS065a, CS081a, CS090a, CS113, CS131, CS147, CS149, CS159, CS207) [35, 36]*.* The other 4 strains of Actinobacteria were *Verrucosispora* ML1, *Saccharopolyspora erythraea* ATCC11635, *Rhodococcus erythropolis* DSM 43006, and *Micromonospora melanospora* ATCC3104. All strains were grown on agar Medium A (MOPS 21 g, glucose 5 g, yeast extract 0.5 g, meat extract 0.5 g, casaaminoacids 1 g, pH = 7 per liter) [37] plates incubated at 30 ℃ for 7 days. For metabolite production, strains were grown on agar plates of soy flour mannitol (SFM) (soy flour 20 g, mannitol 20 g per liter) [38] and YMA (yeast extract 3 g; malt extract 3 g; peptone 5 g and glucose 10 g per liter). [34].

### Actinobacteria co-culture

**Fig. 1:**
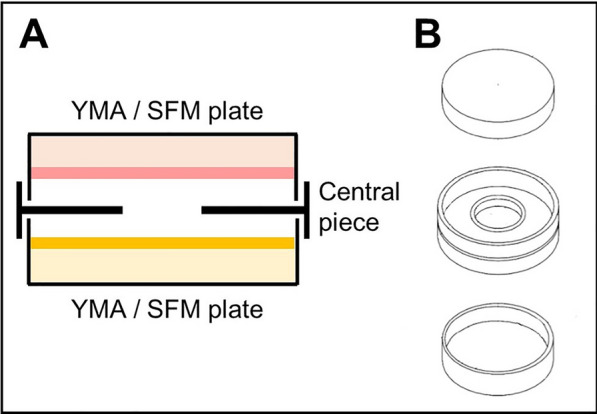
VOC chamber. **A** Schematic representation of a VOC chamber. **B** Overview of each part of the VOC chamber device. The hole in the middle allows the exchange of VOCs between cultures (modified from [39])

Actinobacteria strains were grown on SFM or YMA plates at 30 ℃. A 20% glycerol solution of each Actinobacteria was streaked all over the plates using a sterile loop. Glycerol solutions had been previously quantified, so the same amount of spores from each bacteria was added. After 24 h of the growth of these Actinobacteria, the VOC chamber was assembled (J.D. Catalán S.L., Arganda del Rey, Madrid, Spain), which consisted of a central piece that allows generating a growing chamber for both microorganisms and the exchange of volatiles between the two species tested. This device was mounted with the one-day-old grown bacteria plates (one facing up and the other facing down) as shown in Fig. 1 [34]. In this way, both cultures exchange volatile compounds with each other through the hole of this device, without any physical contact between both microorganisms or with the compounds that diffuse into the medium. Streptomycete and non-streptomycete microorganisms were placed in the bottom or top part without distinction as there was demonstrated no correlation between the observed effect and the place where each bacteria was placed (data not shown). The chamber was sealed with Parafilm^®^ (Bemis, E-Thermo Fisher Scientific, Madrid, Spain) to avoid the breakout of volatile compounds and incubated at 30 ℃ for 6 days. The combinations used in this co-cultures were each of the 11 streptomycete strains with *Micromonospora* sp*., Saccharopolyspora* sp*., Verrucosispora* sp*.* and *Rhodococcus* sp*.*, and also each of these last four Actinobacteria with each other. Monocultures of the different species were used as control samples. Each experiment was made in triplicate.

### Secondary metabolites extraction and chromatography analysis

3.5 g (fresh weight) of the plates (agar+microorganism) was extracted with ethyl acetate (VWR Chemicals), acidic ethyl acetate containing 1% formic acid (ThermoFisher), or butanol (Sigma-Aldrich). After 1–2 h of mixing with solvents in a shaker at 200 rpm at room temperature, the organic phase was collected and dried under vacuum. Subsequently, the dried extracts were resuspended in 200 µL methanol and 10 µL of samples were injected into the UPLC. Samples were run on an Acquity UPLC I-Class (Waters) using a BEH C18 column (1.7 μm particle size, 2.1 mm × 100 mm) employing acetonitrile and water containing 0.1% of trifluoroacetic acid as mobile phase. A gradient was used from 10 to 99% of acetonitrile in 10 min and a flow rate of 0.5 mL/min. For HPLC analysis, a Waters ZQ4000 system was used connected to an HPLC 2695/2795. An Alliance chromatographic system coupled to a SunFire C18 column (3.5 μm particle size, 2.1 mm × 150 mm) and a 996 PDA detector was employed. Acetonitrile and MQ water + formic acid 0.1% were used as mobile phase and elution was performed with an isocratic hold with acetonitrile (10%) for 4 min followed by a linear gradient of acetonitrile (10–88%) over 30 min (0.25 mL/min). Empower 3.0 program was used to compare and analyze the chromatograms obtained from each sample. The comparison between chromatograms was qualitative.

### Dereplication

HRMS-based compound dereplication was performed in Medina Foundation using their in-house library and the Dictionary of Natural Products version 26:2 to identify already known compounds. The retention time, together with the exact mass and the derived molecular formula was used as criteria to search in databases. An Agilent 1200 Rapid Resolution HPLC coupled with a maXis Bruker qTOF mass spectrometer was used. The volume injected was two µL and a Zorbax SB-C8 column (2.1 × 30 mm, 3.5 µm particle size) was used for separation. The mobile phase consisted of solvent A, 90:10 milliQ water-acetonitrile, and solvent B, milliQ water-acetonitrile, both with 13 mM ammonium formate and 0.01 TFA. Samples were eluted with a 0.3 mL/min flow rate, and the gradient used was 90% to 0% to solvent A/10% to 100% solvent B in 6 min, 0% solvent A/100% solvent B in 2 min, 0% to 90% solvent A/10% to 100% solvent B in 0.1 min, and 90% solvent A/10% solvent B for 9.1 min. The maXisqTOF mass spectrometer was operated in ESI-positive mode. Source conditions were 4-kV capillary voltage, end plate offset = 500 V, dry gas (N2) flow = 11 L/min; dry temperature = 200 ℃, and nebulizer (N2) pressure at 2.8 bars.

### Bioactivity analysis

Bioassays against *Micrococcus luteus* (Gram-positive bacteria), *Escherichia coli* (Gram-negative bacteria), and the yeast *Candida albicans* were performed to test antibiotic production of the cultures using agar plates of TSA (agar Tryptic Soy Broth) (for bacteria) and YMA (for the yeast). An agar plug from each grown Actinobacteria culture was placed on top of the bioassay plate. Likewise, solvent-extracted samples (from the 3,5 g) were resuspended in methanol and 20 µL of each sample was added into a diffusion bioassay disc. Also controls with methanol were used in order to make sure the activity was not resultingof the solvent effect. The plates were finally incubated for 16 h at 30 ℃ (antifungal tests) or 37 ℃ (antibacterial tests). Inhibition diameters were measured and compared with the control samples. Each test was performed in triplicate. From these data, the mean and standard deviation of the millimeters of halo were calculated.

## Results

The co-culture of the different strains of Actinomycetes in SFM or YMA, through the exclusive exchange of volatile compounds, has demonstrated the effect that VCs emitted by one bacterial genus can exert on another related genus. Volatile compounds from a neighboring strain can generate metabolic responses in other specific strain of different types. In this assay, we focus on the analysis of responses in secondary metabolism. Our observations are based on chromatographic analysis of metabolites produced under VC exposure versus non-exposure. These analyses were complemented by antibiotic activity tests (bioassays) to find differences in metabolite profile that cannot be detected by our chromatographic system. A visual analysis of the appearance of the coculture plates compared to the corresponding monoculture plates is also carried out in order to detect differences in coloration, appearance, sporulation, growth, etc. A summary of bioassay results is shown in the Additional file Table 1. The results presented here can be divided into four types: (i) no observed effect; (ii) inhibition of the production of some metabolites; (iii) overproduction of compounds already produced by the bacteria without confrontation to VCs from other bacteria and, (iv) production of new metabolites not produced by the bacteria in monoculture, due to the awakening of the related biosynthetic pathway by the exposure to VCs. In order to make the article more concise, only the most relevant chromatographic comparisons are shown. The compilation of all the results obtained can be seen in the Additional file Tables 2–12.

### Effects observed on the different* Streptomyces* species after exposure to non-streptomycete VCs (ns-VCs).

#### *Streptomyces* sp. CS014

**Fig. 2:**
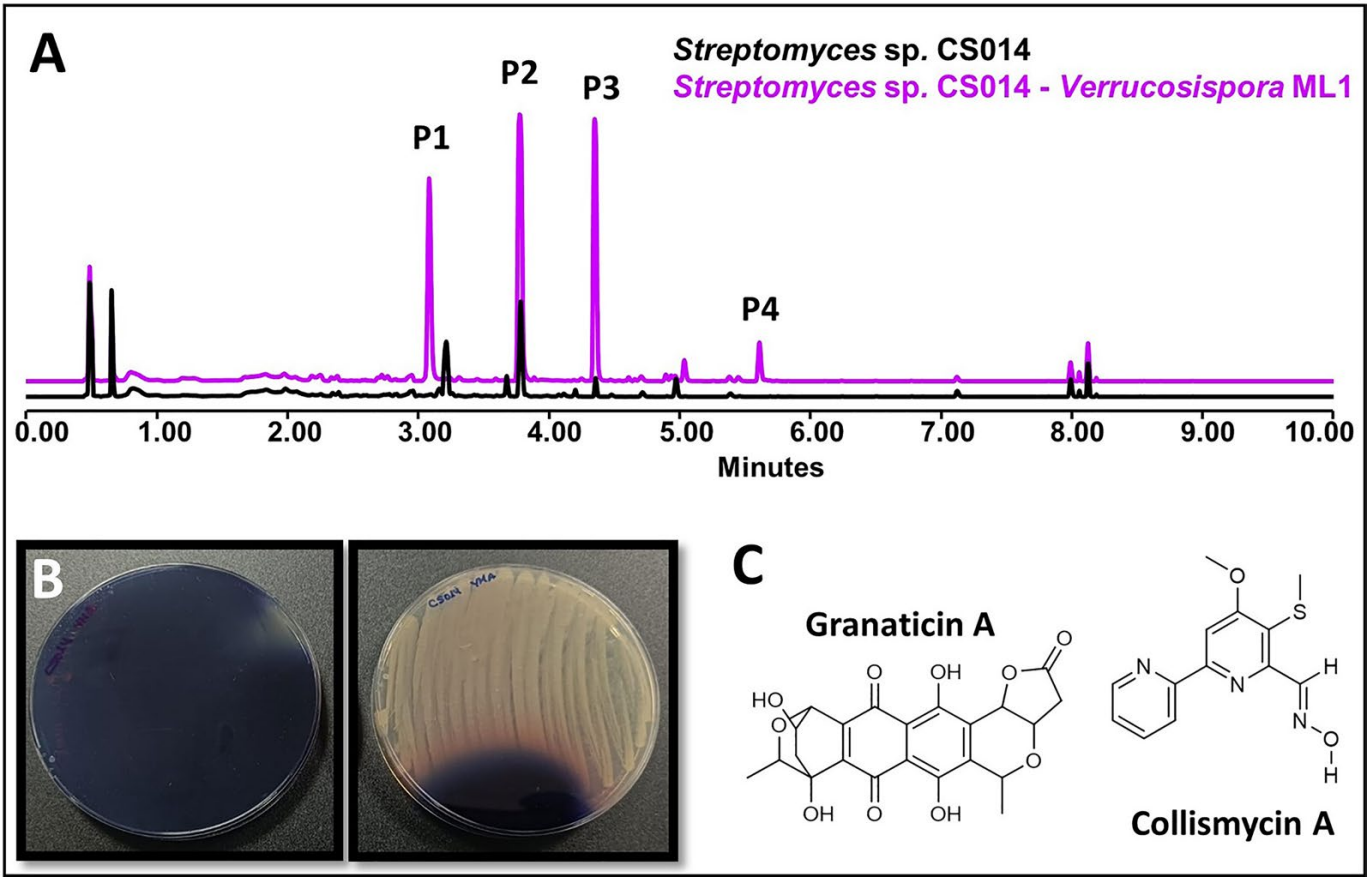
Co-culture *Streptomyces* sp. CS014 against *Verrucosispora* ML1 in YMA medium. **A** Comparative UPLC profile of CS014 cultured on YMA against *Verrucosispora* ML1 and extracted with ethyl acetate. P1-4 show the overproduction of granaticins A and C and the activation of the synthesis of collismycins A and D. P1 = collismycins A-B, P2 = granaticin A, P3 = granaticin C, P4 = collismycin D. **B** *Streptomyces* sp. CS014 plates after co-culture against *Verrucosispora* ML1 (left) and in monoculture (right)*.* Due to the blue color of granaticins, the production of this metabolite can be appreciated visually. **C** Structure of granaticin A and collismycin A

The coculture of *Streptomyces* CS014 against different non-streptomycete actinomycetes using VCs interactions has resulted in a greater modification of the streptomycete metabolism when this strain was cocultured in YMA medium. In SFM medium, only the overproduction of granaticins A and C was detected in coculture with *M. melanospora*. No appreciable differences were observed in the rest of the co-cultures in this medium. In YMA, more changes in chromatographic profiles are observed than in the SFM medium: in all cocultures, an overproduction of granaticins A and C (Fig. 2A, C). Also in YMA, an overproduction of other metabolites can be observed after exposure to ns-VCs, such as aloesaporin, cyclo (tyr-pro) and N-acetyltyramine, and the activation of collismycins A and D. Given the blue color of granaticins, the overproduction of this compound generates noticeable visual differences (Fig. 2B). It is worth highlighting differences in bioassay against *M. luteus*when samples of the strain were co-cultured against *R. erythropolis* on YMA medium. This resultswere shown both in the bioassays in form of agar plugs (Additional fileTable 1, Additional file Fig. 1) and the ones with the extracts of the three solvents used (acetate, ethyl acetate containing 1% formic acid, butanol). An outcome of the results obtained in the co-cultures of this strain is shown in the Additional file Table 2.

#### *Streptomyces* sp. CS057

**Fig. 3:**
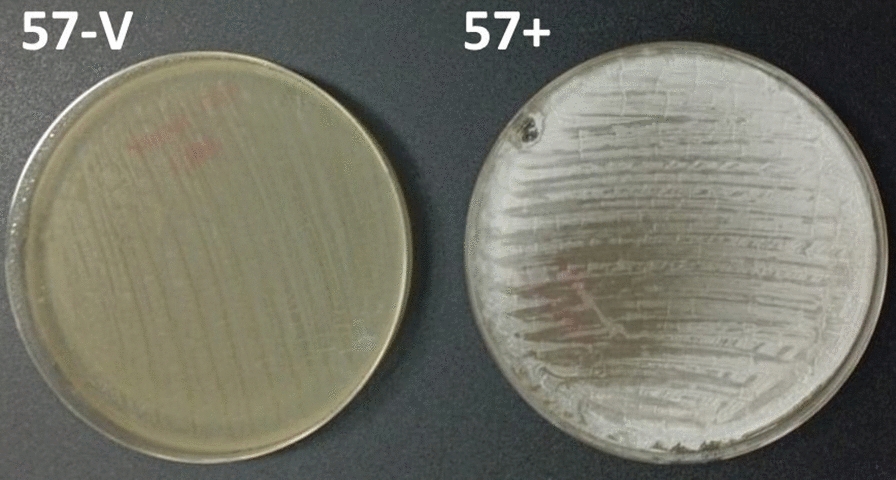
Plate of *Streptomyces* sp. CS057 in co-culture against *Verrucosispora* ML1 (left) and in monoculture (right) in YMA medium. It is shown that, when *Streptomyces* sp. CS057 is co-cultured with *Verrucosispora* ML1, its sporulation capacity is lost

The coculture of *Streptomyces* CS057 strain with other non-streptomycete actinomycetes in exclusive interaction with VCs resulted in different metabolic responses depending on the medium used and the strain in coculture. The co-culture of *R. erythropolis* and *Streptomyces* sp. CS057 showed an overproduction of cycloheximide in both media tested. Butanol and ethyl acetate with 1% formic acid extracts of the cultures against *S. erythraea* displayed coproporphyrins and cycloheximide overproduction in SFM and YMA, respectively. Cultures against *Verrucosispora* sp. in SFM and *M. melanospora* in YMA present an overproduction of actiphenol. The most interesting result was the activation of the production of skyllamicins A and B observed in samples obtained from *Streptomyces* sp. CS057 after exposure to ns-VCs (except *R. erythropolis*) in SFM medium (Additional file Table 3).Curiously, in co-culture with *Verrucosispora* ML1, the streptomycete results in a poor sporulation capacity that is visible (Fig. 3). Although in this work we detected other visual changes such as color changes, all of these are due to changes in the production of metabolites. This change observed in the *Streptomyces* CS057 strain is the only one in which a change in the sporulation capacity is detected. This invites us to think about other effects that VCs can exert, not only affecting the secondary metabolism.

#### *Streptomyces* sp. CS065a

**Fig. 4:**
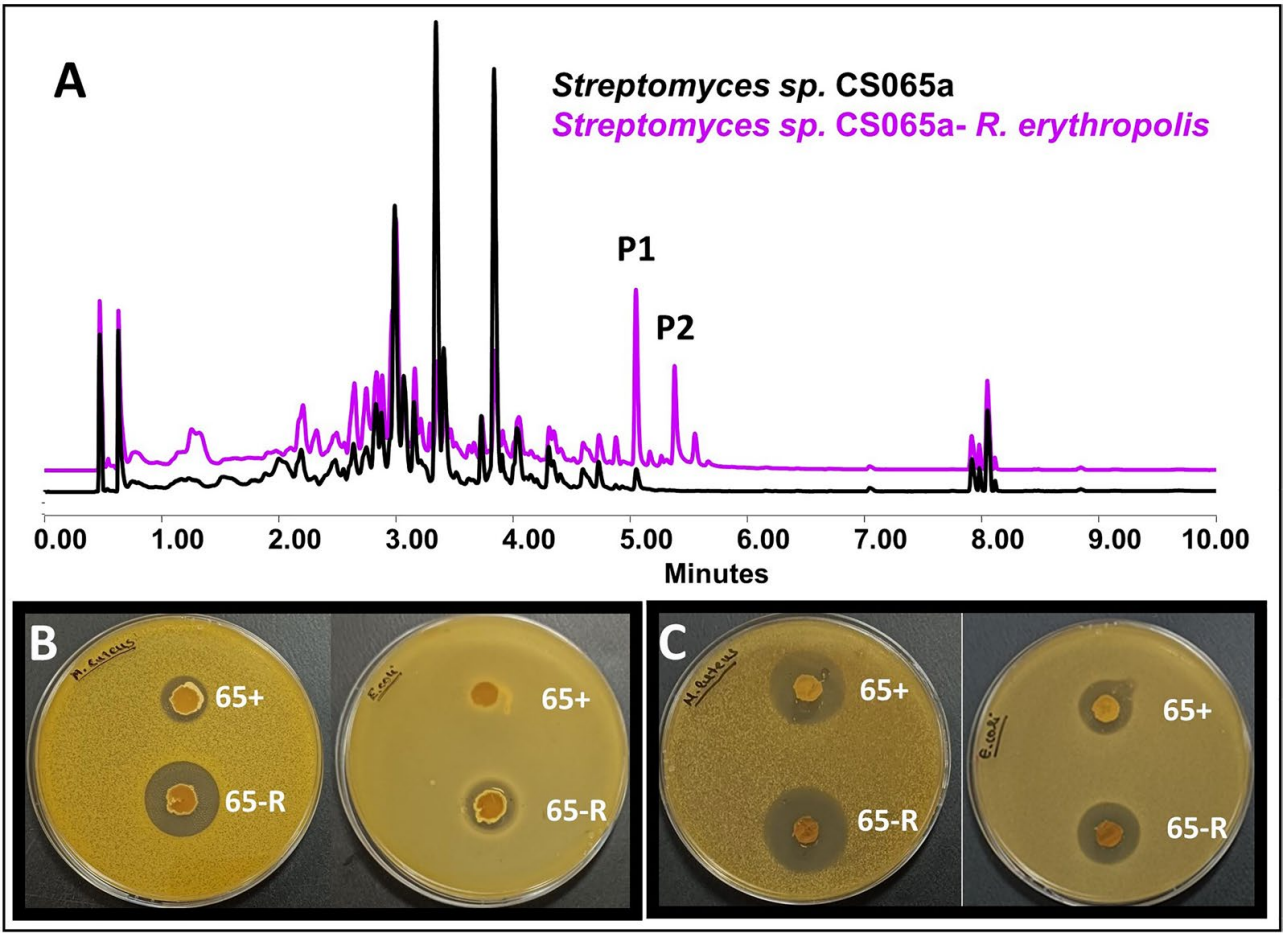
Co-culture *Streptomyces* sp. CS065a against *R. erythropolis* in SFM medium. **A** Comparative UPLC profile of CS065a against *R. erythropolis* and extracted with ethyl acetate containing 1% formic acid, pointing to the overproduction of chromomycins (P1) and the activation of alteramides production (P2). **B** Bioassay from the SFM agar plug of the monoculture *Streptomyces* sp. CS065a (65 +) and the co-culture against *R. erythropolis* (65-R) against *M. luteus* (left, 65 +  = 15 ± 2 mm inhibition zone; 65-R = 26 ± 2.52 mm inhibition zone) and *E. coli* (*luteus* (right, 65 +  = 0 ± 0 mm inhibition zone; 65-R = 16 ± 0.57 mm inhibition zone). **C** Bioassay from the YMA agar plug of the monoculture *Streptomyces* sp. CS065a (65 +) and the co-culture against *R. erythropolis* (65-R) against *M. luteus* (left, 65 +  = 23 ± 1.52 mm inhibition zone; 65-R = 26 ± 0.57 mm inhibition zone) and *E. coli* (right, 65 +  = 16 ± 1.15 mm inhibition zone; 65-R = 19 ± 2.30 mm inhibition zone)

**Fig. 5:**
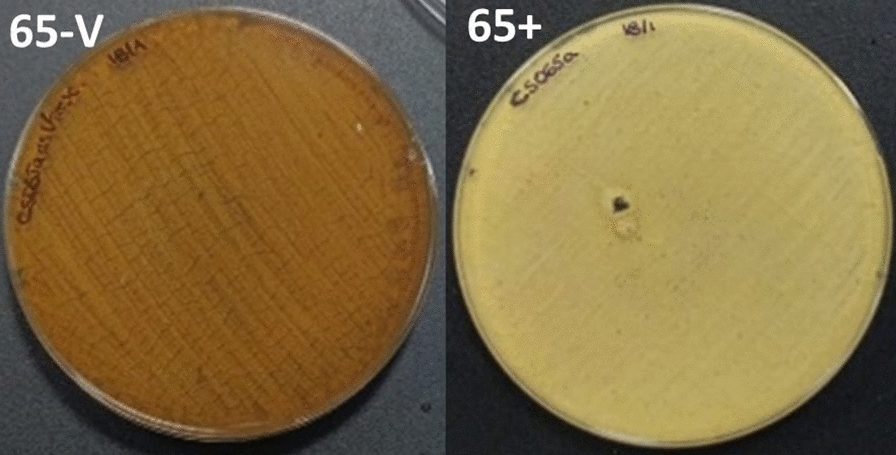
Activation of chromomycin production in *Streptomyces* sp. CS065a. Plate of *Streptomyces* sp. CS065a in co-culture against *Verrucosispora* ML1 (left) and in monoculture (right) in YMA medium. Due to the brown color of chromomycins, the production of this metabolite can be appreciated by the change of color

Coculture trials of this strain together with other non-streptomycete actinomycetes have resulted in different metabolic changes, highlighting the activation of synthesis of alteramides (in some assays) (Fig. 4A) and the overproduction of chromomycins (in all trials), which involved a brown color change in the plates that can be visually appreciated (Fig. 5). The Actinomycete co-cultures not only result in remarkable metabolic changes in *Streptomyces* sp. CS065a, but also in the generation of inhibition halos in bioassay (Additional file Table 1). Bioassays against *M. luteus* and *E. coli*, both by the agar plugs and by the extract samples (Fig. 4B, C), showed an increment in bioactivity when exposed to *R. erythropolis* VCs. Alteramide and chromomycins production can be responsible for the effects observed against *M. luteus,* however, these metabolites lack activity against Gram-negatives. The compound(s) responsible for the differential activity against *E. coli* are unknown probably because those bioactive compounds cannot be detected under our analytical test conditions. Interestingly, also a negative effect on metabolite production was observed after exposure to VCs in some co-cultures. A resume of the results is shown in Additional file Table 4.

#### *Streptomyces* sp. CS081a

**Fig. 6:**
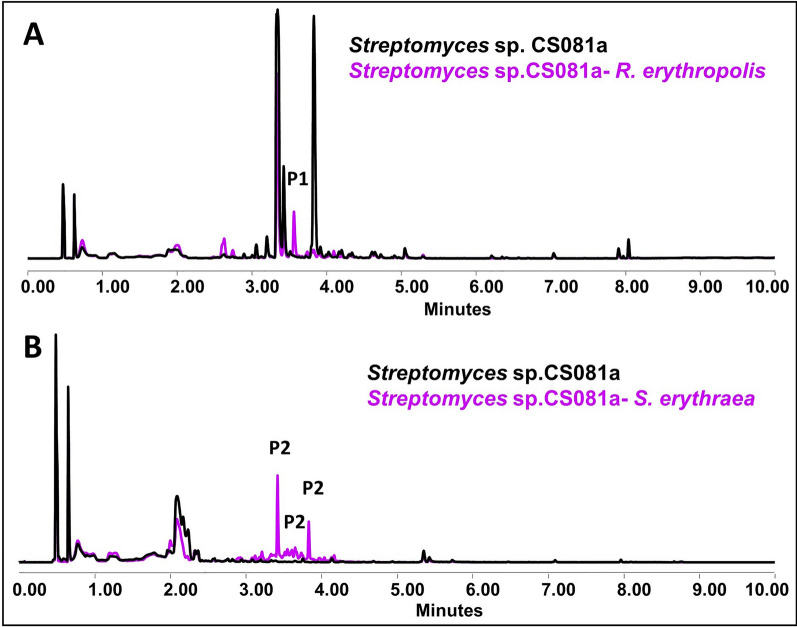
Cosmomycin production in *Streptomyces* sp. CS081a. **A** Comparative UPLC profile extracted with ethyl acetate of *Streptomyces* sp. CS081a against *R. erythropolis*. P1 indicates the overproduction of dihydrotetrodecamycin. **B** Comparative UPLC profile extracted with ethyl acetate of *Streptomyces* sp. CS081a against *S. erythraea*. P2 indicates the activation of the production of cosmomycin-related compounds

Coculture of the *Streptomyces* CS081a strain against non-streptomycetes actinomycetes produced different metabolic changes. When this strain is co-cultured in SFM medium against *M. melanospora, S. erythraea,* and *Verruscosispora* ML1, no appreciable differences are shown either in the bioassays or in the respective chromatographic profiles. Only the coculture in this medium against *R. erythropolis* produced a metabolic change, an overproduction of dihydrotetrodecamycin (Fig. 6A). a result that was also repeated in this confrontation in YMA medium. However, when this strain is exposed to ns-VCs in YMA, an activation of cosmomycin production can be observed (Fig. 6B). As also occurs with the previous species, a decrease in the production of some unidentified compounds is observed after exposure to certain ns-VCs. An overview of the results is shown in the Additional file Table 5.

#### *Streptomyces* sp. CS090a

**Fig. 7:**
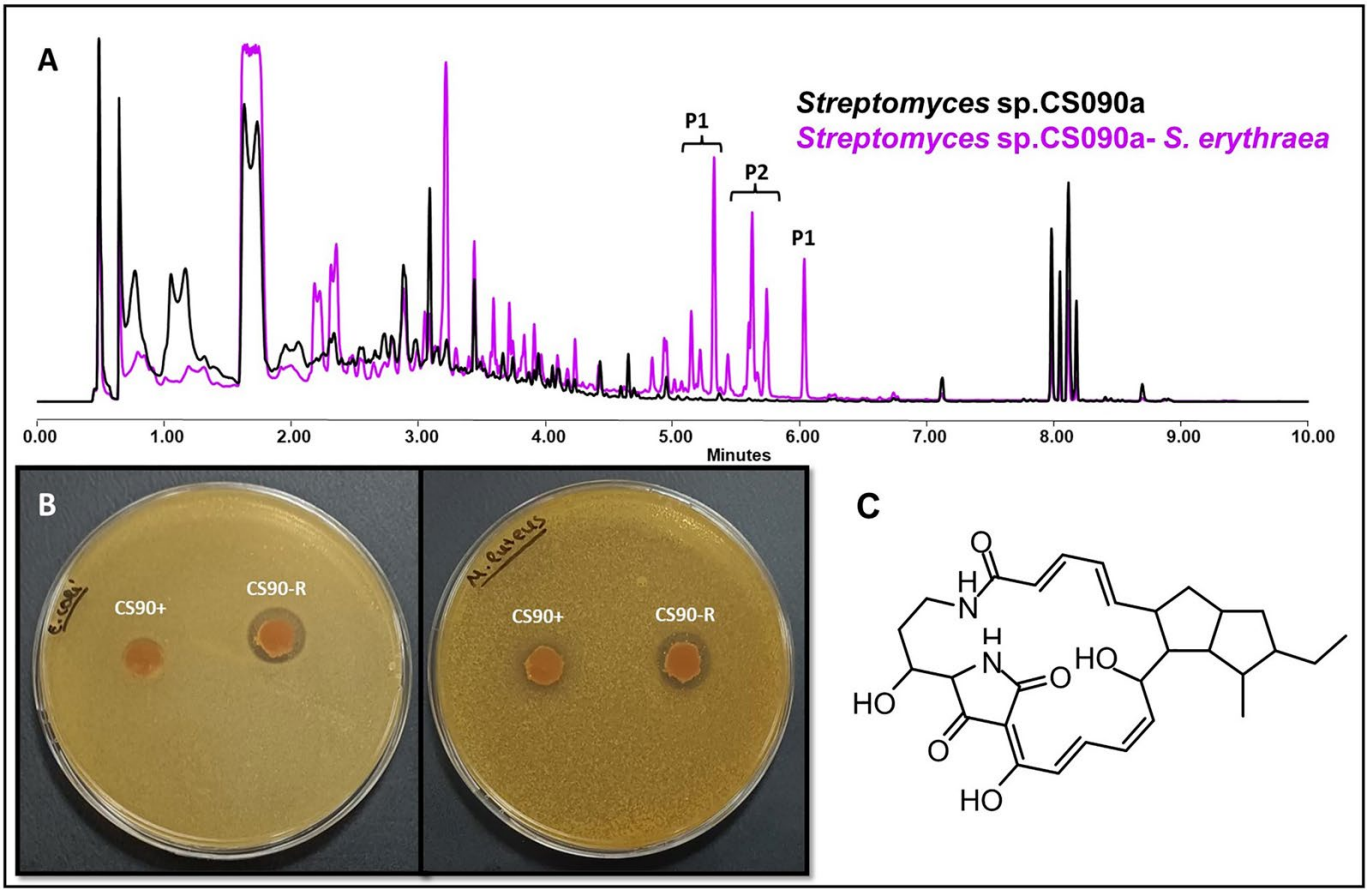
Alteramide activation in *Streptomyces* sp. CS090a. **A** Comparative UPLC profile *of Streptomyces* sp. CS090a cultured on SFM against *S. erythraea* and extracted with ethyl acetate 1% formic acid. P1 and P2 show the activation of the synthesis of maltophilins and alteramides, respectively. **B** Bioassay plate against *E. coli* (left) and *M. luteus* (right) with samples obtained from *Streptomyces* sp. CS090a cultured in SFM against *R. erythropolis*. The inhibition zone against *E. coli* is 0 ± 0 for control (CS90 +) and 15 ± 1.73 mm for CS090a co-cultured (CS90-R). The diameter inhibition against *M. luteus* is 9 ± 0.57 mm for control (CS90 +) and 13 ± 1.15 mm for *Streptomyces* sp. CS090a co-cultured (CS90-R). **C** Chemical structure of alteramides

Overproduction and activation of bioactive secondary metabolism pathways can be seen when *Streptomyces* sp. CS090a grows in several co-cultures. In this sense, the overproduction of 2-aminobenzoic and the activation of alteramides and maltophilins synthesis isobserved in YMA co-cultures (except in the presence of *Verrucosispora* ML1) (Fig. 7A). Although it cannot be attributed to any specific compound, it has been observed that on the bioassays of the SFM co-cultures against *R. erythropolis* differential inhibition zone against *E. coli* are shown. The latter culture also shows an increased activity against *M. luteus* that can be the consequence of the alteramide activation (Fig. 7B). Again, the production of some unidentified metabolites decreased after exposure to certain ns-VCs. A resume of the results is presented in the Additional file Table 6.

#### *Streptomyces* sp. CS113

**Fig. 8:**
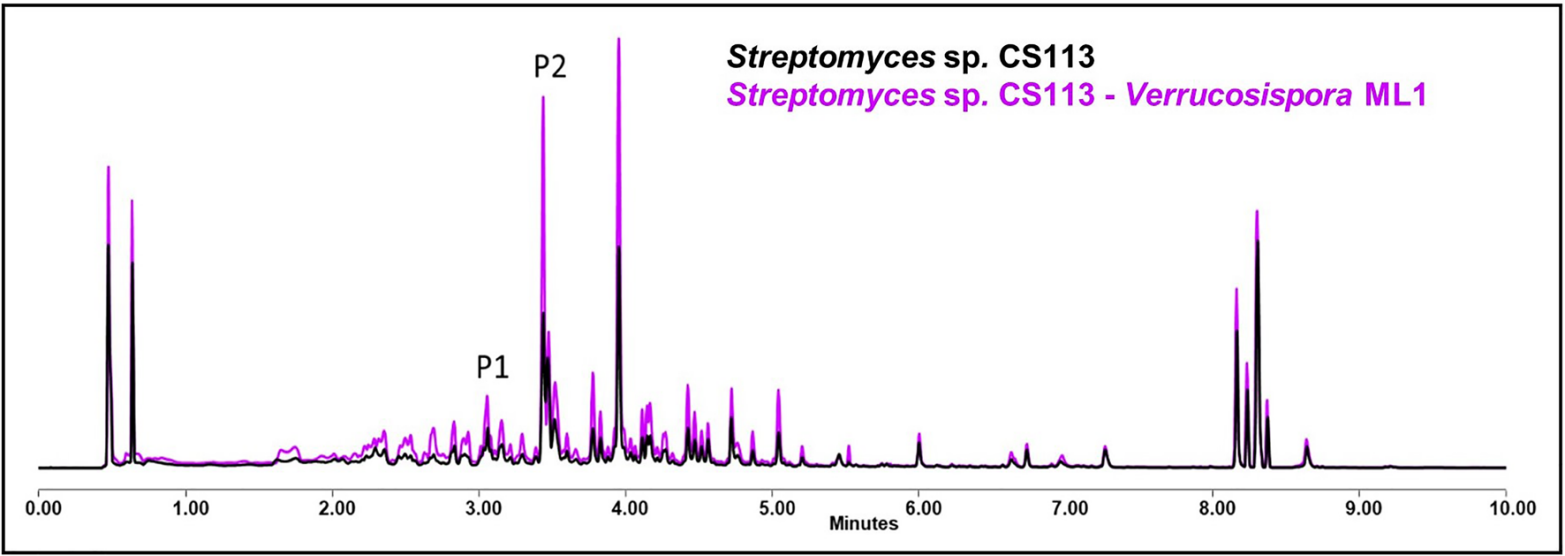
Comparative UPLC profile of *Streptomyces* sp. CS113 against *Verrucosispora* ML1 and extracted with ethyl acetate. P1 indicates the overproduction of germicidin and P2 the overproduction of cervimycin

The overproduction of germicide by *Streptomyces* sp. CS113 can be seen after exposure to several ns-VCs (Fig. 8), as well as the increase in the yield of coproporphyrins, daidzein, and cervimycin in other specific cases. In general, a greater increase is observed when *Streptomyces* sp. CS113 is grown in SFM, unlike the other *Streptomyces* strains where most of them showed an improved metabolite production in YMA. A compilation of the results is shown in the Additional file Table 7.

#### *Streptomyces* sp. CS131

**Fig. 9:**
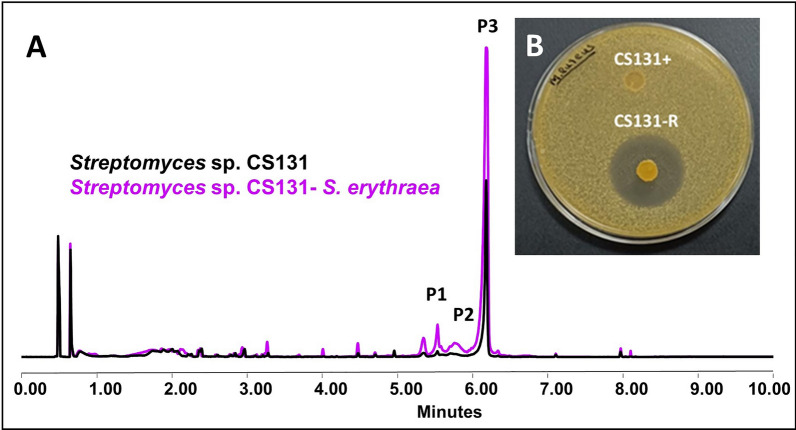
Results of the co-culture *Streptomyces* sp. CS131—*S. erythraea* sp. **A** Comparative UPLC profile of *Streptomyces* sp. CS131 cultured on YMA against *S. erythraea* and extracted with ethyl acetate. P1 indicates the overproduction of actinomycin G4, P2 the activation of actinomycin I synthesis and P3 the overproduction of actinomycin D. **B** Bioassay plate against *M. luteus* when microorganisms are cultured in SFM. CS131 +corresponds with the control monoculture while CS131-R stands for the agar plug from the co-culture against R. *erythropolis.* An inhibition zone of 30 ± 1.15 mm of diameter is produced by CS131-R sample while there is no antibacterial activity exerted by the metabolites from the control agar plug

In cocultures with this strain it has only been identified variations in different forms of the actinomycin metabolite. In *Streptomyces* sp. CS131, no major changes were perceived in the chromatographic profile when co-cultured in the presence of *Verrucosispora* ML1 or *M. melanospora*. However, the overproduction of actinomycin D is observed in co-culture with *S. erythraea* in both media and with *R. erythropolis* in SFM, as well as the overproduction of the actinomycin G4 and the activation of the synthesis of actinomycin I after exposure to ns-VCs from *S. erythrea* in YMA (Fig. 9A). Regarding the bioassays, a differential inhibition zone against *M. luteus* was detected from the plug agar of this strain when it was co-cultured with *R. erythropolis* in SFM (Fig. 9B). Given that the control (monoculture) did not generate inhibition, we hypothesize that some sort of a metabolic pathway activation occurs. However, as no new differential peaks were detected in the chromatographic profile, we have not identified the possible compound(s) responsible for this effect, which seems cannot be extracted with the organic solvents used. A summary of the results is shown in the Additional file Table 8.

#### *Streptomyces* sp. CS147

**Fig. 10:**
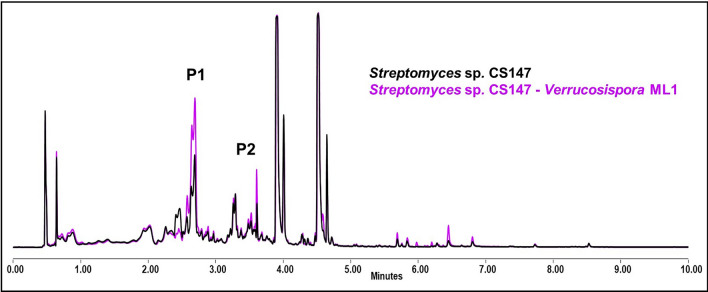
Comparative UPLC profile of *Streptomyces* sp. CS147 cultured on SFM against *Verrucosispora* ML1. P1 indicates the overproduction of cyclo (pro-leu) and P2 the overproduction of coproporphyrin

*Streptomyces* sp. CS147 shows a slight yield increase of vicenistatin when growing in YMA (data not shown). In addition, overproduction of cyclo (leu-pro) and coproporhyrin occurs when grown in SFM in the presence of *Verrucosispora* ML1 (Fig. 10). No differences on bioassays were observed. An outcome of the results is shown in the Additional file Table 9.

#### *Streptomyces* sp. CS149

**Fig. 11:**
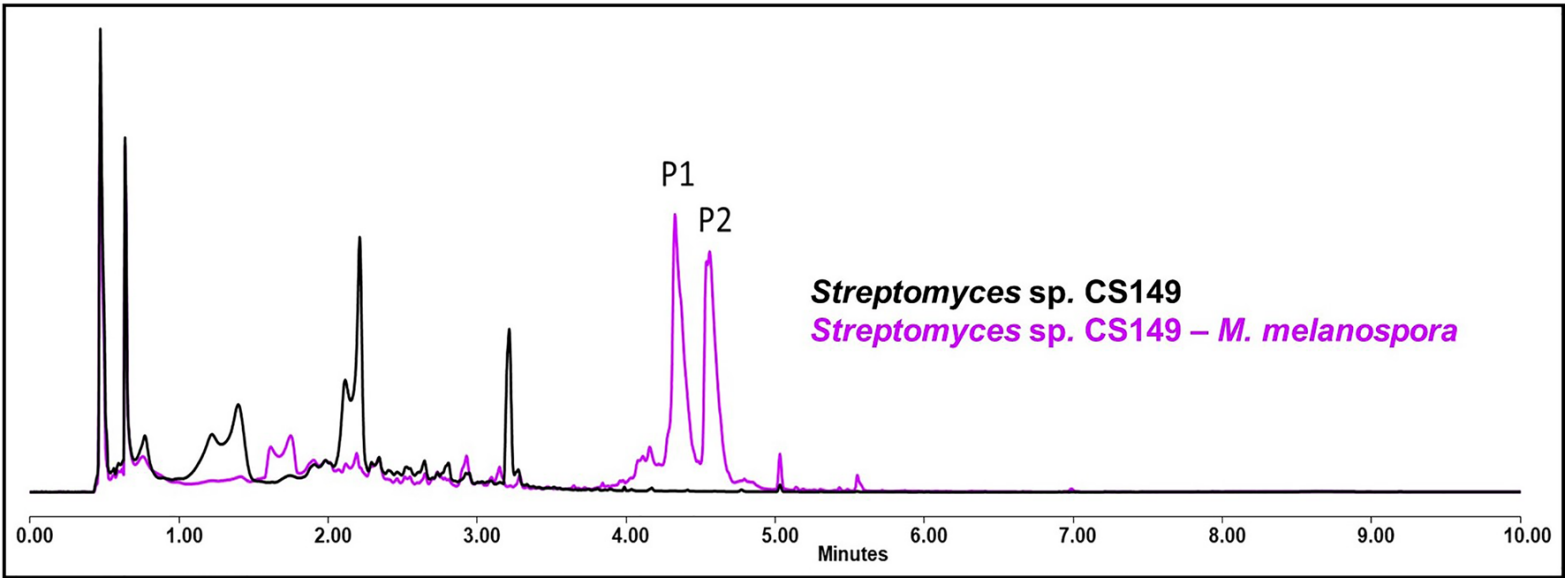
Comparative UPLC profile of *Streptomyces* sp. CS149 cultured on YMA against *M. melanospora*. P1 and P2 indicate the activation of rumycin 1 and rumycin 2 biosynthesis, respectively

One of the most remarkable activations of a silent biosynthetic pathway by the effect of ns-VCs during this work was observed in *Streptomyces* sp. CS149 co-cultured in YMA with *M. melanospora* (only observed in the butanol extract). De novo biosynthesis of rumicins 1 and 2 was triggered only after exposure to ns-VCs (Fig. 11) and these compounds can be responsible for the increased bioactivity of the butanol extracts of the co-culture against *M. luteus*. Also, the overproduction of collismycins in the SFM co-culture in the presence of *S. erythraea* was detected. As already observed in *Streptomyces* sp. CS065a, CS081a and CS090a species, there is a reduction in the generation of some compounds after exposure to ns-VCs. A summary of the results is shown in the Additional file Table 10.

#### *Streptomyces* sp. CS159

**Fig. 12:**
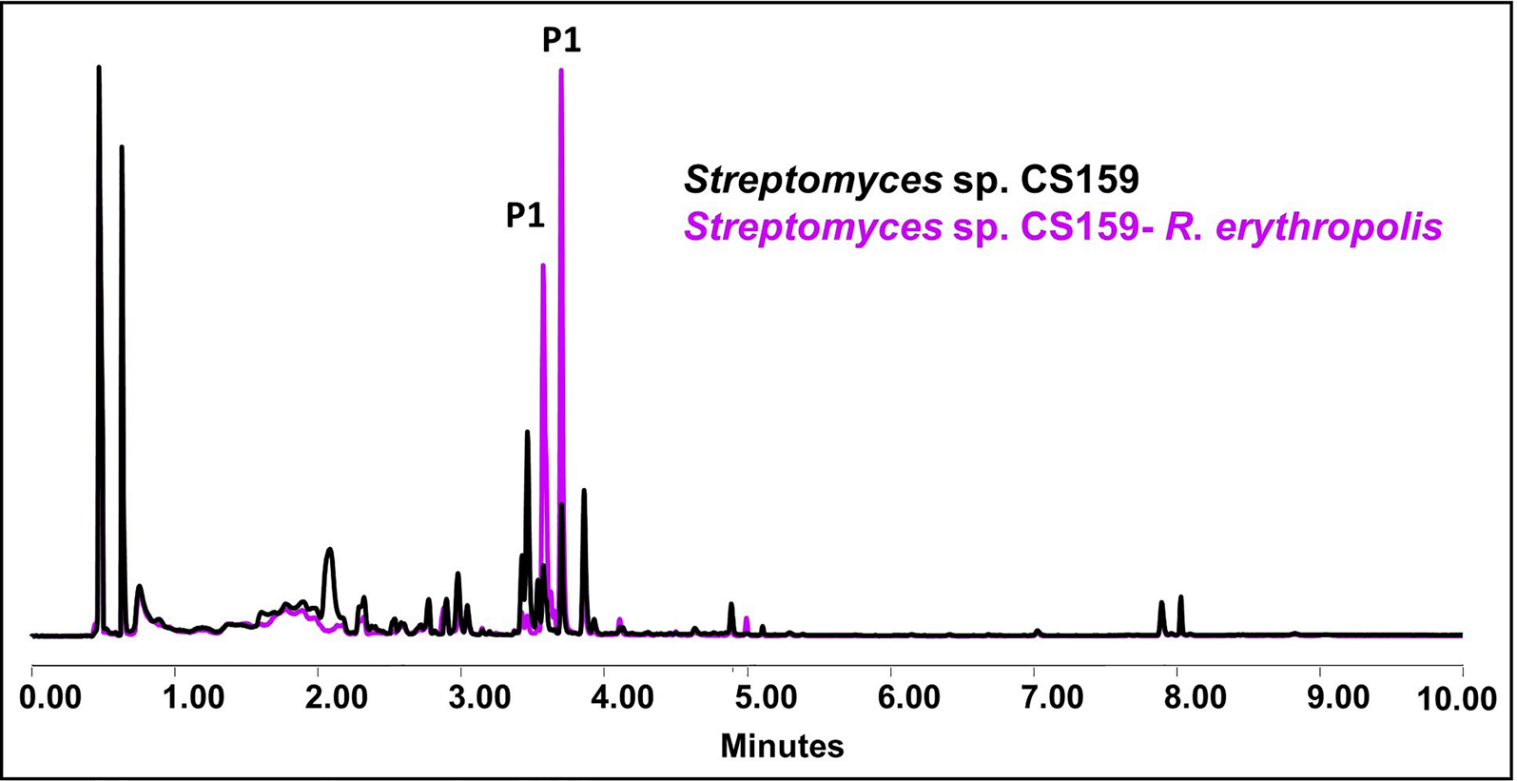
Comparative UPLC profile of *Streptomyces* sp. CS159 cultured on YMA against *R. erythropolis*. P1 indicates the overproduction of inthomycins

The only metabolic change detected in *Streptomyces* sp. CS159 is the overproduction of inthomycins exposed to ns-VCs from *R. erythropolis* in YMA (Fig. 12) and *S. erythraea* in SFM. No differences on bioassays were observed. An outcome of the results is shown in the Additional file Table 11.

#### *Streptomyces* sp. CS207

**Fig. 13:**
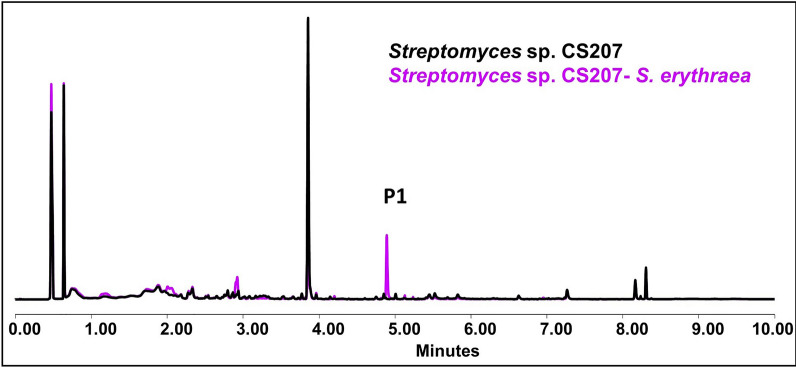
Comparative UPLC profile of *Streptomyces* sp. CS207 cultured on YMA against *R. erythropolis*. P1 indicates the overproduction of 3-cyanomethyl-6-prenylindole

Increased production of various prenylidol derivatives such as 3-(2-hydroxyethyl)-6-prenylindole and 3-cyanomethyl-6-prenylindole was spotted in *Streptomyces* sp. CS207 after exposure to ns-VCs (Fig. 13). No differences on bioassays were observed**.** A summary of the results is shown in the Additional file Table 12.

### Effects observed on non-streptomycete Actinobacteria after exposure to VCs produced by *Streptomyces* species (s-VCs).

#### *R. erythropolis* DSM43006

More differences are seen in the YMA medium than in the SFM culture, although the production of metabolites is greater in the latter. In general, the changes are very similar regardless of the confronted strain. In all cases, the activation of the biosynthesis and the increase in production of yet unidentified compounds are detected. The most general increased production is observed in co-culture with *Streptomyces* sp. CS159 (Additional file Fig. 2). No antibiotic activity could be assigned to the new metabolites produced after s-VCs exposure.

#### *S. erythraea* ATCC11635

The co-culture in SFM medium does not show appreciable differences in liquid chromatography, while culture in YMA medium implies the overproduction of certain secondary metabolites that lack identification by dereplication (Additional file Fig. 3). In the bioassay test, no zones of inhibition were observed either by the monocultures or in the co-cultures, so this strain does not produce compounds with antibiotic qualities or at least active against the microorganisms tested.

#### *Verrucosispora* ML1

**Fig. 14:**
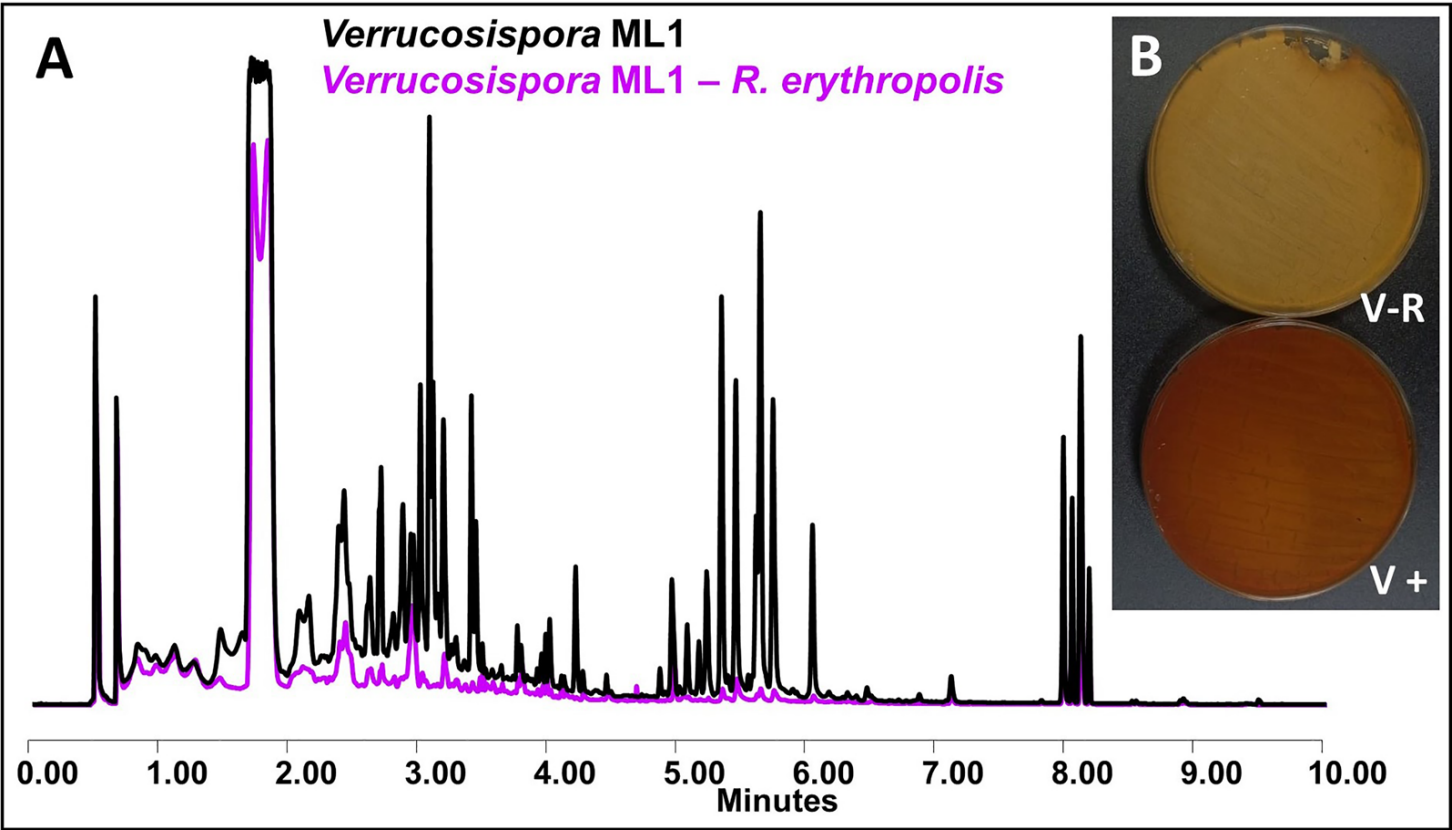
Results of the co-culture of *Verrucosispora* ML1.—*R. erythropolis* DSM43006 (**A**) Comparative UPLC profile of *Verrucosispora* ML1. Cultured on YMA against *R. erythropolis* DSM43006 and extracted with ethyl acetate containing 1% formic acid. It can be appreciated the inhibition of the production of several metabolites. **B** Plate of *Verrucosispora* ML1 in co-culture against *R. erythropolis* DSM43006 (left) and in monoculture (right) in YMA medium. Due to the inhibition of the production of several metabolites, differences can be appreciated visually

This Actinomycete shows increased production at different levels both in co-culture with *Streptomyces* sp. and with other Actinobacteria with one exception: when this strain is co-cultured with *R. erythropolis* DSM43006 in YMA medium an inhibition of the production of certain metabolites occurs (Fig. 14).

#### *M. melanospora* ATCC3104

In different co-cultures with *Streptomyces* species, *M. melanospora* shows increased production of different compounds which could not be identified by dereplication (Additional file Fig. 4). However, co-cultures with the other Actinobacteria it did not show changes in the metabolic profile.

## Discussion

Actinobacteria phylum is one of the most diverse in nature with representatives showing a variety of morphologies, many of them with complex life cycles and widely distributed both in aquatic and terrestrial environments [21]. This great diversity is reflected in their secondary metabolism possibilities since the sequencing of their genomes has revealed they are potential producers of great versatility of bioactive metabolites [40]. The group of *Actinobacteria* has been highly exploited at an industrial level due to the interest aroused by the multiple properties of these compounds [41–43].

Although the majority of BGCs are silent in laboratory conditions, many efforts have been focused on activating these genetic clusters with a variety of techniques [23]. However, at the laboratory level, most of those approaches have been carried out using monocultures of different strains of bacteria or fungi [44]. Given the great need to search for new bioactive compounds, it is necessary to apply creative and different strategies to find novel metabolites despite the success obtained in activating BGCs with such approaches, it must not be overlooked that many of the microbial metabolites of interest are produced for a specific activity [44–47]. Indeed, the role of microbial interactions in the activation of metabolic responses is noteworthy, and among them is the production of metabolites of interest [44].

In this work, we have studied the effect of volatile compounds as inducing agents since it is not a widely exploited strategy as the use of diffusible compounds given the difficulty of manipulation of such metabolites, their limit of detection, and as there are no widely standardized analytical methods [46–48] To do so, we have confronted 15 different Actinobacteria to VCs produced by each other and analyze the metabolic changes, in terms of secondary metabolite production, that may happen. The assays shown above have been performed using volatile exchange chambers that allow the bidirectional flow of these messengers without physical contact between the microorganisms or with the compounds that diffuse into the medium. This means that the effects evaluated in this work are exclusively due to volatile compounds and can thus be differentiated from the ones by diffusible compounds.

To this extent, the aim is to demonstrate how the use of cocultures, and specifically those mediated by VCs, is an efficient non-targeted strategy to activate silenced metabolic pathways in other microorganisms as well as to increase the production of compounds, which also arouses interest to industrial level. Therefore, we show how the commonly used strategy of cocultures can be modified based solely on VC, such that these compounds stimulate secondary metabolism responses and generate new products of interest. The chambers used in this experiment had been used on other occasions for analysis of interaction by volatiles in other species such as fungi-bacteria [49], fungi-fungi [39], fungi-insect [50], or fungi-plant [51]; so this essay is the analysis of a more specific type of interaction, between organisms of the same phylum.

### Changes in sporulation capacity

The differences in the sporulation capacity of the different cocultured strains compared to those in the monoculture were visually observed. With the exception of *Rhodococcus erythropolis,* the other species tested in this work are spore-forming, a form of resistance generated under certain environmental stresses that highlights the complex life cycle of Actinomycetes [52]. Looking closer, the life cycle of Actinomycetes in culture surfaces harbors a complex signaling network [53], which is summarized in the germination of a spore, development of substrate mycelium, consisting of long multinucleated hyphae¸ and subsequently specialized aerial mycelium that eventually differentiate into spores chains when conditions are not satisfactory [54, 55].

Only in one case were notable differences in sporulation capacity identified: when *Streptomyces* sp. CS057 is co-cultured with *Verrucosispora* ML1it can be observed as a "bald" phenotype which means this strain is not capable of sporulation when exposed to *Verrucosispora* ML1volatiles (Fig. 3). However, we consider that it is not truly a bald phenotype given that most mutants that lack the ability to form aerial hyphae are usually deficient in the production of secondary metabolites [56]. In our case, the production pattern of the strain is not affected, so the observed effect does not correspond to a permanent affection on the development of the aerial mycelium. Although we do not know at what point one or more of the volatile compounds of the emitting strain affected *Streptomyces* sp. CS057, the result translates into an inability to sporulate which can be perceived visually without affecting metabolic production.

In other works, volatile compounds produced by actinomycetes have been shown to inhibit mycelial growth and consequently metabolite production [57]. However, on that occasion what is affected is exclusively the formation of the spore and not the development and growth of the hyphae or the metabolic production, so in our case, we suggest that the VCs might affect the group of *whi* genes since it has been previously reported that *Streptomyces* strains are capable of forming hyphae but do not sporulate [58].

Suppression of the sporulation capability has also been observed in other species: VCs produced by *Bacillus velezensis* prevent the fungus *Botrytis cinerea* spore formation [59, 60]. This fact highlighted the importance of the signaling effect of VCs not only between closely related species but even at the interkingdom level. Additionally, VCs such as 2-methyl-2-butene produced by *Rhizopus arrhizus* inhibit spore germination in a cell density-dependent manner [61]. Similarly, volatiles of *B. amyloliquefaciens* NJN-6 also hinder spore germination [62]. In this case, it is unknown which VC is responsible for the effect nor at what level of the signaling pathways it affect, but it is deduced that the inhibition is not found at the level of spore germination but at the level of formation. There is no extensive recent bibliography providing volatile candidates that can generate this effect, although the scientific community have been aware of the mentioned consequences for a long time since articles such as Garrett and Robinson [63] point to nonanoic acid as an inhibitor of spore formation [63].

### Increased metabolite production

In a previous work [34], we have already described how volatiles emitted by members of the same genus can trigger changes in the metabolism of other microorganisms co-cultured with them. In the present case, by co-culturing different strains of Actinobacteria we observed how the effect is maintained even though these groups no longer belong to the same genus, but they are still phylogenetically close bacteria. In general, the results are dependent on the strain, culture media, and the companionship strain present in the co-culture, however, we observed, as a consequence of co-culture, the production of certain metabolites increased.

[48]. The production of secondary metabolites is indeed higher in *Streptomyces* spp. than in other phyla-sharers, for example in *Rhodoccocus*, where there is hardly any production of secondary metabolites in laboratory conditions. For that reason, we expose here how the metabolism of these bacteria is much more variable and results more affected in co-culture than in other Actinobacteria. *S. erythraea,* previously classified as *Streptomyces erythraeus*, is also a great producer of a large variety of compounds. Because this species is closer to *Streptomyces* than *Micromonospora, Verrucosispora,* and *Rhodococcus*, it is inevitable to consider that the variability of compounds that it can produce is also closer to the metabolic profiles offered by streptomycetes, with abundant secondary metabolites.

We have observed how there are certain metabolites and, accordingly, the genes involved in their biosynthesis, that are most likely to be influenced in some way by the presence of VCs. For example, in *Streptomyces* sp. CS014 the production of granaticins was increased in all the co-cultures evaluated, effect perceived visually since the culture acquires a violet hue tone (Fig. 2). However, other compounds only see their production increase after exposure to VCs from another specific microorganism; for example, in *Streptomyces* sp. CS113 there was only an overproduction of cervimycin when co-cultured alongside *Verrucosispora* ML1 (Fig. 8). These findings might imply the existence of VC inducers that have a more global action and activate different silenced metabolic pathways or that several Actinobacteria produce the same volatile that generates the observed effect [34].

In general, regardless of the microorganisms, the YMA medium is better than SFM in order to observe these metabolic changes, probably because the production of volatile compounds that serve as an agent that modifies the metabolism is much more favored in that particular medium due to the different raw materials that they contain (Table 1 shows an overview of all the results obtained during this work). For the SFM medium, no significant changes were obtained in 43.18% of the trials, while in YMA this percentage drops to 27.2% of the experiments. These statements are based on the experiments that are included in this work and in previous ones [34], where we observed greater differences in metabolism profiles when the cocultures were performed in YMA medium than in others (i.e. SFM and R5A). For the present work, we discarded the utilization of R5A [65] given the low positive results observed in previous reports [34]. YMA and SFM have no components in common. Both are complex media since they contain elements without exact composition such as yeast extract, malt extract, or soy flour. Also, YMA medium has peptone which is an important source of nitrogen. The R5A medium is diametrically opposite since it contains numerous ingredients and has various oligoelements and a pH of 6.8. Some of these compounds in R5A are common with the YMA medium; glucose in the same proportion (10 g/L) and yeast extract in 5 g/L in R5A compared to 3 g/L in YMA. However, neither of these two compounds are determinants in the production of volatiles that activate metabolic pathways, since the differences in effects observed between them are very notable. As future projections to clarify this point, a comparative GC–MS analysis of the production profile of volatile compounds in each medium should be performed.

**Table 1:**
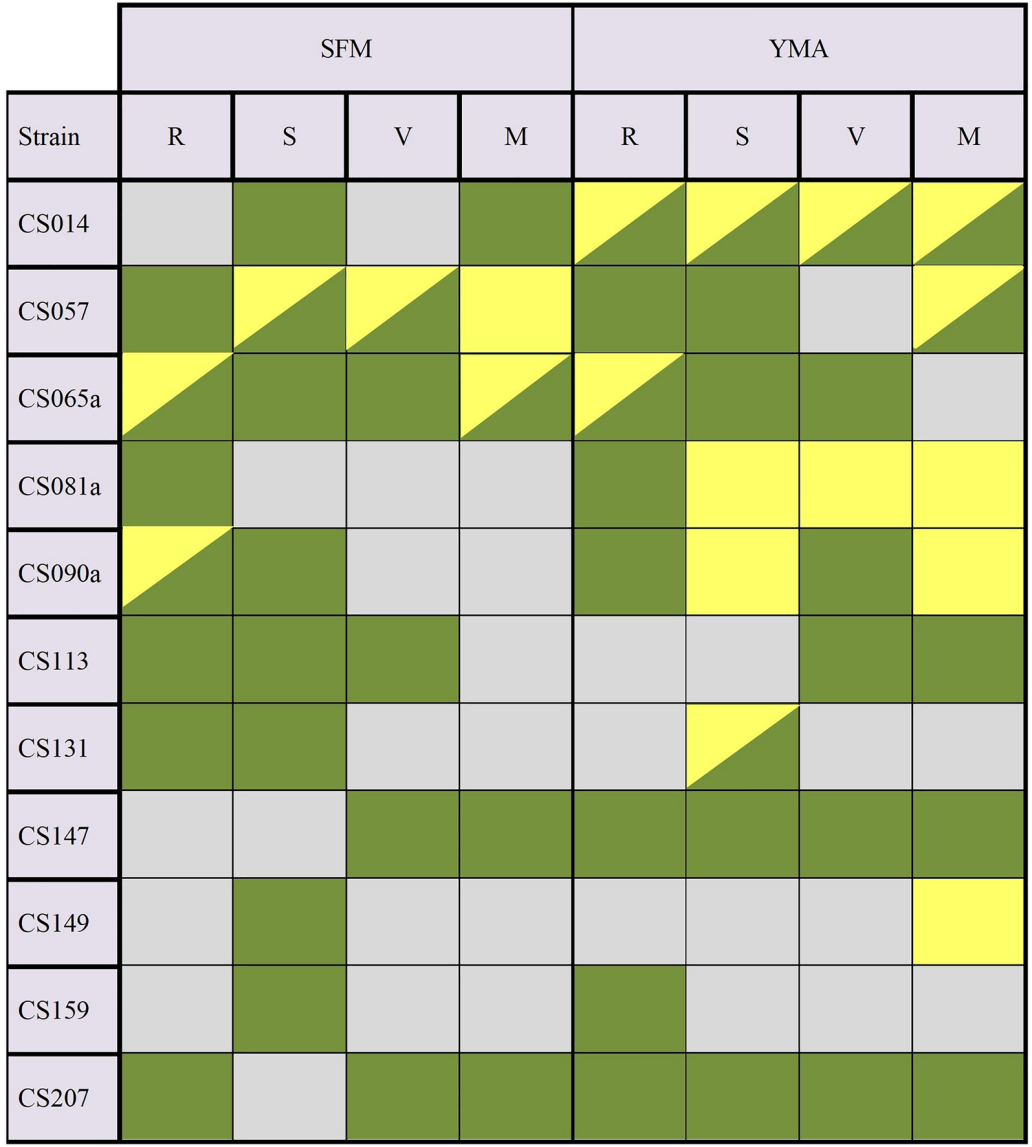
Summary of the effects observed on the *Streptomyces* sp. strains as a consequence of the co-cultures with different Actinobacteria compared with the cultures of isolated streptomycetes

Green color indicates metabolite overproduction; yellow indicates new metabolite production and grey no remarkable differences. R = *R. erythropolis* DSM 43006; S = *S. erythraea* ATCC11635; V = *Verrucosispora* ML1; M = *M. melanospora* ATCC3104

It is worth mentioning, that in previous works it has already been described how volatile compounds can generate variations in the growth and/or metabolism of other organisms, not necessarily belonging to the same genus or even sharing a phylum. Such as those inter-species relationships described between bacteria and fungi, bacteria and plants, or bacteria and animals [3, 66, 67].

### Metabolite synthesis activation, A source of drug discovery?

Similarly to that discussed in the previous section, silenced biosynthetic pathways get activated because of VCs present in co-cultures. Unfortunately, the frequency of activation events is not very high leading to the lack of novel compounds identified during our analytical approaches. This fact highlights the great problem faced by new drug development and discovery programs that constantly rediscover already known compounds [68–70]. At present, new compounds are being discovered less and less often using traditional techniques (e.g. OSMAC); for that reason, modern techniques are applied, but they require a large amount of time and involve a high economic cost [70]. In this work, a novel technique has been used, using volatile compounds interactions to activate silent clusters. This approach has not been used as much compared to classical cocultures in liquid media. This technique allows a non-specific approach since it can produce effects in different BGCs (non-targeted screening). Differences were observed in the metabolic profile of strains in coculture compared to wild-type, and an attempt was made to identify these metabolites. Unfortunately, not all of them could be identified, probably due to limitations in the methodology performed. One of the great limitations is the deconvolution process since it is difficult to identify compounds without background information and from complex mixtures [71]. An efficient method seems to be used by microorganisms that share niches in these cultures, an approach which was already evaluated in [49] since it seems reasonable that if the subjects under study share a niche they are likely to compete with each other. Communication between individuals living in the same environment has a fundamental role in the genesis of behavioral changes due to variations in gene transcription. In these environments, different relationships of competition, mutualism, and symbiosis are established. Under laboratory conditions there is a lack of the aforementioned interactions so it is necessary to simulate these conditions, to some extent to observe specific responses [64, 72]. There also is a possibility that the unidentified compounds are undescribed metabolites, however further studies on this topic are necessary.

In this work, fifteen Actinobacteria strains, among which there are 11 *Streptomyces* spp., have been co-cultivated in two different media and a large number of differences have been observed as a consequence of the effect of VCs from the companion organism: differences in sporulation, activation of silenced pathways, increased production of compounds, etc. Although not all of these Actinobacteria were isolated from the same niche, they are phylogenetically related and all of them present enormous metabolic potential [18, 19, 73–76]. However, the results have not shown any new unknown compounds. Interestingly, in the case of species *Streptomyces* sp. CS065a and CS131 the activation of a bioactive compound production was evidenced by bioassay but it cannot be detected under our chromatographic analysis. The results obtained in the bioassay tests, summarized in the Additional file Table 1, show how all cases in which there are differences in the bioassays occur when the strains are co-cultured with *R. erythropolis.* One of the reasons could be that the methodology is not appropriate (in terms of solvent extraction and/or analytical method). In future work, we will extend this screening by using different organic solvents for extraction and different analytical techniques (e.g. GC–MS). As an example of drug rediscovery, we have rumycins 1 and 2 that we previously managed to activate their biosynthesis in *Streptomyces* sp. CS149 in the presence of VCs produced by *Streptomyces* sp. CS131 or CS081a species [34]. In the present case, under the same conditions (YMA medium and extraction with butanol), the activation of rumycins is observed when the strain is co-cultured with *M. melanospora*. In turn, it suggests that both *Streptomyces* sp*.* strains CS131, CS081a, and *M. melanospora* produce the same or a similar mixture of VCs that induce the same effect.

Although most of the effects observed in this work because of VC exposure are in a positive sense (increased production of metabolites or activation of new metabolic pathways), interactions were also observed in a negative direction such as inhibition or decrease in production. This is the case of the co-culture between the Actinobacteria *Verrucosispora* ML1 and *R. erythropolis* where we observe reduced production of *Verrucosispora* metabolites, which is noticeable in the chromatographic profile as well as visually in the differential coloring of the culture plate (Fig. 14). This could be an example of a response mechanism to competitive relationships, where one organism inhibits the production of secondary metabolites of another and therefore it is left at a disadvantage by lacking defense tools [77].

Despite the cons observed during the development of this work, to exploit effect of the VCs by co-culturing *Streptomyces* spp. alongside non-streptomycete Actinobacteria is still a valid approach to identifying novel bioactive compounds, mainly considering the great metabolic potential of the entire group of Actinobacterias [20, 73]. Specifically, some *Verrucosispora* spp. produce compounds with antitumor activity such as harrucomycin C and cyperusol C, or antibacterial activity such as Brevianamide F or (2-(hydroxymethyl)-3-(2-(hydroxymethyl)-3-methylaziridin-1-yl) (2-hydroxyphenyl) methanone [78–80]. On the other side, *S. erythraea* is well known for the production of erythromycin, *Rhodococcus* spp. produce antibiotics such as rhodostreptomycin A (*R. fascians* 307CO) and the polyketide auraquine RE (*R. erythropolis* JCM 6824), or siderophores such as rhodobactin (*R. rhodochrous* strain OFS) [18, 74, 76]. In addition, *Micromonospora* spp. generate compounds such as actinomycin C2 or blastomycin, being another Actinobacteria with great production of interesting therapeutic metabolites [75, 81].

That is why, to increase the success of medicines based on natural products discovery, it will be necessary to elucidate the triggers and signals that activate the expression of the genes involved in their biosynthesis. Therefore, although it is outside the scope of this work, it could be a useful tool to design assays more focused on the activation of silenced pathways if potential volatile compounds that induce secondary metabolism are identified. In the end, mimicking natural environments puts the microorganisms to the limit, forcing them to behave as they would in the natural environment, where they face competitive relationships and other stressors.

## Conclusions

The co-culture of microorganisms is presented as a strategy to exploit the metabolic potential of microorganisms. Given the great need of the pharmaceutical market for an extensive search for new bioactive compounds, mimicking the environment of the microorganisms under controlled conditions stands as an alternative of interest, since it has been shown that this type of cultures differ substantially in metabolites production from monocultures. In this work, we have focused on the signaling effect of volatile compounds in the metabolism of other close-related bacteria. Not only changes have been observed in the metabolism but also changes in growth and sporulation capacity are reported. The exposure of microorganisms to the stress of other competing organisms arises as a fascinating strategy and the use of volatile compounds in these studies highlights the great role that these compounds represent as metabolism-inducing agents. These metabolites are increasingly gaining interest in the scientific community and represent an alternative to the studies of diffusible compounds that are widely developed.

### Supplementary Information

Additional file 1.

## Data Availability

All data generated or analysed during this study are included in this published article [and its supplementary information files].
