# Supplementary material for: Volatile communication in Actinobacteria: a language for secondary metabolism regulation
Source: Microb Cell Fact. 2024 Jun 18;23:181. doi: 10.1186/s12934-024-02456-4 (PMC11186294; doi:10.1186/s12934-024-02456-4)
Supplement: Supplementary file 1 — Additional file 1. [file 12934_2024_2456_MOESM1_ESM.docx]

**Additional file**

Additional Table1: summary of differential results of bioassays

|  | Bioassay against *M. luteus* | Bioassay against *E. coli* |
| --- | --- | --- |
| CS014+ (YMA) | 20±1.52 mm | - |
| CS014 vs *R. erythropolis* (YMA) | 26±1.73 mm | - |
| CS065a+ (SFM) | 15±2 mm | 0 mm ±0 |
| CS065a vs *R. erythropolis* (SFM) | 26±2.52 mm | 16 mm ±0.57 |
| CS065a+ (YMA) | 23±1.52 mm | 16±1.15 mm |
| CS065a vs *R. erythropolis* (YMA) | 26±0.57 mm | 19±2.30 mm |
| CS090a+ (SFM) | 9±0.57 mm | 0±0 |
| CS090a vs *R. erythropolis* (SFM) | 13±1.15 mm | 15±1.73 |
| CS131+ (SFM) | 0±0 mm | - |
| CS131 vs *R. erythropolis* (SFM) | 30±1.15 mm | - |

Additional Table 2: overview of the co-cultures of *Streptomyces* sp. CS014:

| Strain | Media | Outcomes |
| --- | --- | --- |
| *R. erythropolis* DSM 43006 | SFM | No effects observed |
|  | YMA | Overproduction of granaticin A and C, aloesaporin, cyclo (tyr-pro), pyrisulfoxin A and N-acetyltyramine. Activation of collismycins production.  Differences on bioassay against *M. luteus* (Additional Figure 1) |
| *S. erythraea* ATCC11635 | SFM | Overproduction of the synthesis of granaticin C |
|  | YMA | Overproduction of granaticin A and C, cyclo (tyr-pro), cyclo (leu-pro), N-acetyltyramine and aloesaporin. Activation of collismycins production. |
| *Verrucosispora* ML1 | SFM | No effects observed |
|  | YMA | Increased production of granaticin A and C, aloesaponarin II, cyclo (Tyr-Pro) and activation of the synthesis of collismycin A and D and coproporphyrins. Extractions with EtAc + 1% formic acid show great differences |
| *M. melanospora* ATCC3104. | SFM | Overproduction of granaticins A and C |
|  | YMA | Overproduction of granaticin, cyclo (Tyr-Pro) and aloesaporin II. Activation of the production of collismycin A, C, and D. |


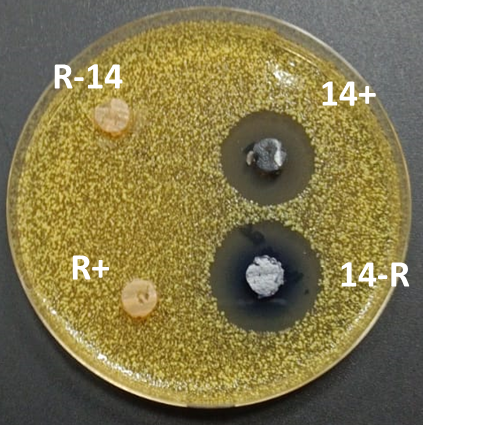


Additional Figure 1: Bioassay from the YMA agar plug of the monoculture *Streptomyces* sp. CS014 (14+) and the co-culture against *R. erythropolis* (14-R) against *M. luteus.* Differences in form of increased halos are shown (14+ = 20 ± 1.52 mm inhibition zone; 14-R = 26 ± 1.73 mm inhibition zone). The lack of activity of the monoculture of *R. erythropolis* (R+) and its coculture against *Streptomyces* sp. CS014 from YMA agar plugs can also be observed.

Additional Table 3: overview of the co-culture of *Streptomyces* sp. CS057.

| Strain | Media | Outcomes |
| --- | --- | --- |
| *R. erythropolis* DSM 43006 | SFM | Overproduction of cycloheximide. Differences on bioassay against bacteria. |
|  | YMA | Overproduction of cycloheximide when metabolites are extracted with butanol. Differences on bioassay against bacteria. |
| *S. erythraea* ATCC11635 | SFM | Overproduction of coproporphyrins and activation of Skyllamycin A and B production when metabolites are extracted with butanol or EtAc with 1% formic acid. |
|  | YMA | Overproduction of cycloheximide |
| *Verrucosispora* ML1 | SFM | Activation of Skyllamycin A and B production, overproduction of actiphenol. Deficiency on sporulation capacity. |
|  | YMA | No differential effects observed |
| *M. melanospora* ATCC3104. | SFM | Activation of Skyllamycin A and B production |
|  | YMA | Overproduction of actiphenol and cycloheximide |

Additional Table 4: overview of the co-culture of *Streptomyces* sp. CS065a.

| Strain | Media | Outcomes |
| --- | --- | --- |
| *R.erythropolis* DSM 43006 | SFM | Increased production of chromomycins and activation of alteramides production. Differences on bioassay against bacteria. |
|  | YMA | Overproduction of chromomycins, 4^A^ ,4^E^ -Dideacetylchromomycin A3, 2. Cyclo([Iso]Leu-Pro), N-acetyltyramine and activation of alteramide production, Differences on bioassay against bacteria. |
| *S. erythraea* ATCC11635 | SFM | Increased production of chromomycins. Differences on bioassay against bacteria |
|  | YMA | Increased production of chromomycins and N-acetyltyramine. Differences on bioassay against bacteria. |
| *Verrucosispora* ML1 | SFM | Increased production of chromomycins. |
|  | YMA | Overproduction of chromomycins. Differences on bioassay against bacteria. |
| *M. melanospora* ATCC3104. | SFM | Activation of alteramide production, overproduction of chromomycins and N-acetyltyramine. Differences on bioassay against bacteria |
|  | YMA | Increased production of chromomycins and activation of alteramides production |

Additional Table 5:overview of the co-culture of *Streptomyces* sp. CS081a.

| Strain | Media | Outcomes |
| --- | --- | --- |
| *R. erythropolis* DSM 43006 | SFM | Increased production of dihydrotetrodecamycin. and cosmomycin |
|  | YMA | Increased production of dihydrotetrodecamycin. |
| *S. erythraea* ATCC11635 | SFM | No differential effects observed |
|  | YMA | Activation of cosmomycin production |
| *Verrucosispora* ML1 | SFM | No differential effects observed |
|  | YMA | Activation of cosmomycin production |
| *M. melanospora* ATCC3104. | SFM | No differential effects observed |
|  | YMA | Activation of cosmomycin and coproporphyrins production |

Additional Table 6: overview of the co-culture of *Streptomyces* sp. CS090a.

| Strain | Media | Outcomes |
| --- | --- | --- |
| *R. erythropolis* DSM 43006 | SFM | Increased production of 2-aminobenzoic. Activation of alteramide production. Differential activity on bioassay |
|  | YMA | Alteramide and maltophilin activation. Differential activity on bioassay. |
| *S. erythraea* ATCC11635 | SFM | Overproduction of 2-aminobenzoic acid |
|  | YMA | Maltophilin and alteramide synthesis activation |
| *Verrucosispora* ML1 | SFM | No differential effects observed |
|  | YMA | Overproduction of 2-aminobenzoic acid |
| *M. melanospora* ATCC3104. | SFM | No differential effects observed |
|  | YMA | Maltophilin and alteramide synthesis activation |

Additional Table 7: overview of the co-culture of *Streptomyces* sp. CS113.

| Strain | Media | Outcomes |
| --- | --- | --- |
| *R. erythropolis* DSM 43006 | SFM | Overproduction of coproporphyrins and germicindin. |
|  | YMA | No differential effects observed |
| *S. erythraea* ATCC11635 | SFM | Increased production of daidzein and germicindin |
|  | YMA | No differential effects observed |
| *Verrucosispora* ML1 | SFM | Overproduction of germicidin |
|  | YMA | Overproduction of germicidin and cervimycin |
| *M. melanospora* ATCC3104. | SFM | No differential effects observed |
|  | YMA | Overproduction of germicicidin |

Additional Table 8: overview of the co-culture of *Streptomyces* sp. CS131.

| Strain | Media | Outcomes |
| --- | --- | --- |
| *R. erythropolis* DSM 43006 | SFM | Actinomycin D overproduction |
|  | YMA | No differential effects observed |
| *S. erythraea* ATCC11635 | SFM | Actinomycin D overproduction |
|  | YMA | Overproduction of actinomycin D and G4. Activation of the synthesis of actinomycin I. |
| *Verrucosispora* ML1 | SFM | No differential effects observed |
|  | YMA | No differential effects observed |
| *M. melanospora* ATCC3104. | SFM | No differential effects observed |
|  | YMA | No differential effects observed |

Additional Table 9: overview of the co-culture of *Streptomyces* sp. CS147.

| Strain | Media | Outcomes |
| --- | --- | --- |
| *R. erythropolis* DSM 43006 | SFM | No differential effects observed |
|  | YMA | Overproduction of vicenistatin |
| *S. erythraea* ATCC11635 | SFM | No differential effects observed |
|  | YMA | Overproduction of vicenistatin |
| *Verrucosispora* ML1 | SFM | Overproduction of cyclo (Leu-Pro) and coproporphyrins |
|  | YMA | Overproduction of vicenistatin |
| *M. melanospora* ATCC3104. | SFM | Overproduction of vicenistatin and cyclo (Leu-Pro) |
|  | YMA | Overproduction of vicenistatin |

Additional Table 10: overview of the co-culture of *Streptomyces* sp. CS149.

| Strain | Media | Outcomes |
| --- | --- | --- |
| *R. erythropolis* DSM 43006 | SFM | No differential effects observed |
|  | YMA | No differential effects observed |
| *S. erythraea* ATCC11635 | SFM | Overproduction of collismycin |
|  | YMA | No differential effects observed |
| *Verrucosispora* ML1 | SFM | No differential effects observed |
|  | YMA | No differential effects observed |
| *M. melanospora* ATCC3104. | SFM | No differential effects observed |
|  | YMA | Activation of rumycin 1 and 2 synthesis (only butanol extraction). Differences on bioassay against *M. luteus* |

Additional Table 11: overview of the co-culture of *Streptomyces* sp. CS159.

| Strain | Media | Outcomes |
| --- | --- | --- |
| *R. erythropolis* DSM 43006 | SFM | No differential effects observed |
|  | YMA | Overproduction of inthomycins |
| *S. erythraea* ATCC11635 | SFM | Overproduction of inthomycins |
|  | YMA | No differential effects observed |
| *Verrucosispora* ML1 | SFM | No differential effects observed |
|  | YMA | No differential effects observed |
| *M. melanospora* ATCC3104. | SFM | No differential effects observed |
|  | YMA | No differential effects observed |

Additional Table 12: overview of the co-culture of *Streptomyces* sp. CS207.

| Strain | Media | Outcomes |
| --- | --- | --- |
| *R. erythropolis* DSM 43006 | SFM | Overproduction of prenylidole derivates |
|  | YMA | Overproduction of prenylidole derivates |
| *S. erythraea* ATCC11635 | SFM | No differential effects observed |
|  | YMA | Increased production of coproporphyrins and prenylidole derivates |
| *Verrucosispora* ML1 | SFM | No differential effects observed |
|  | YMA | Overproduction of prenylidole derivates |
| *M. melanospora* ATCC3104. | SFM | Overproduction of prenylidole derivate |
|  | YMA | Overproduction of prenylidole derivate |


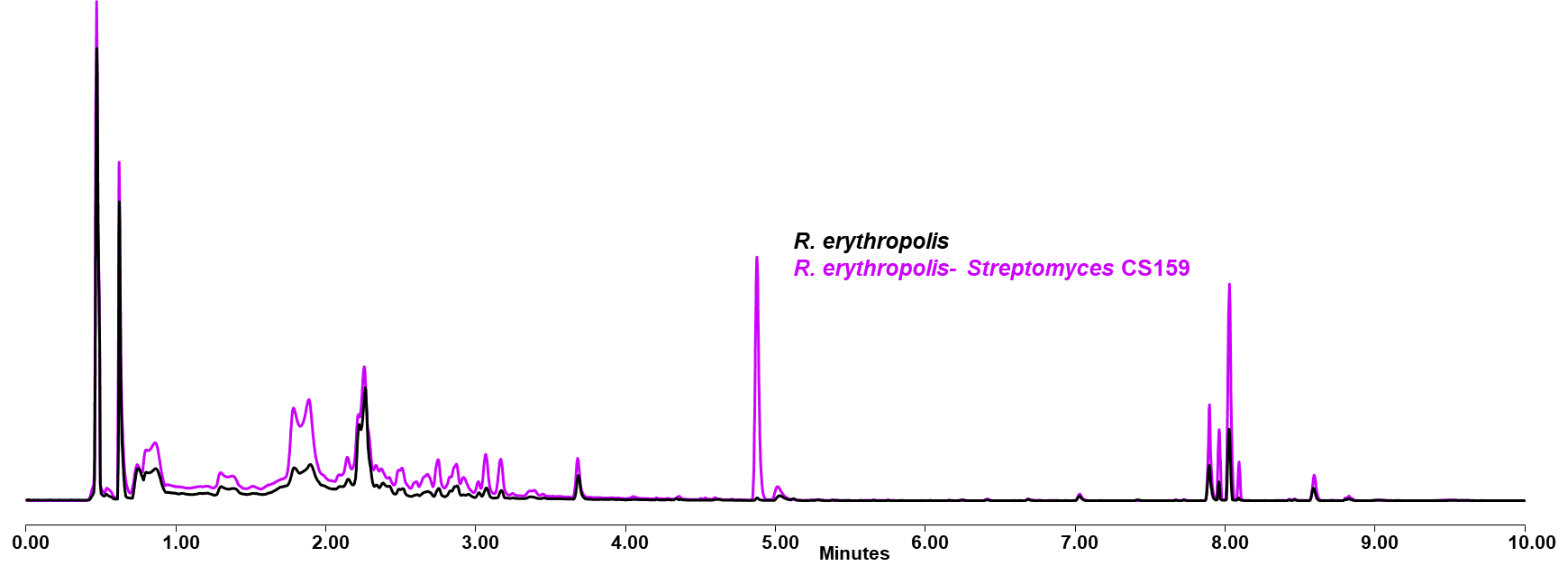


Additional Figure 2: Comparative UPLC profile of *R. erythropolis* DSM43006 cultured on YMA against *Streptomyces* sp. CS159 and extracted with ethyl acetate containing 1% formic acid. It is shown the overproduction and the *de novo* biosynthesis of several metabolites.


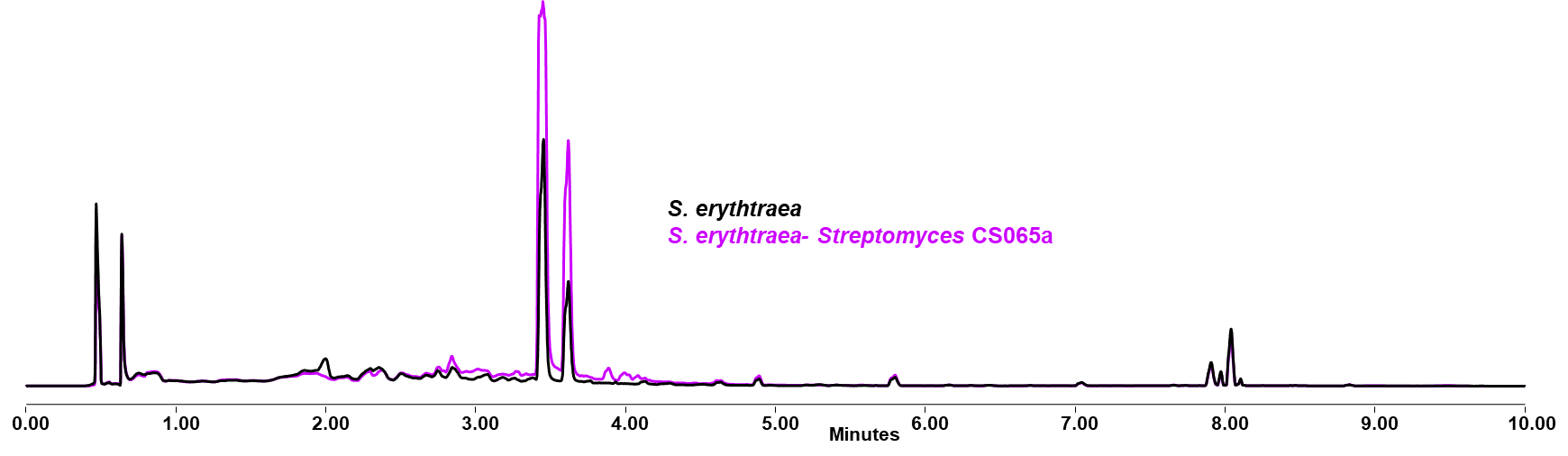


Additional Figure 3: Comparative UPLC profile of *S. erythraea* ATCC11635 cultured on YMA against *Streptomyces* sp. CS065a and extracted with ethyl acetate containing 1% formic acid. It can be appreciated the overproduction of several metabolites.


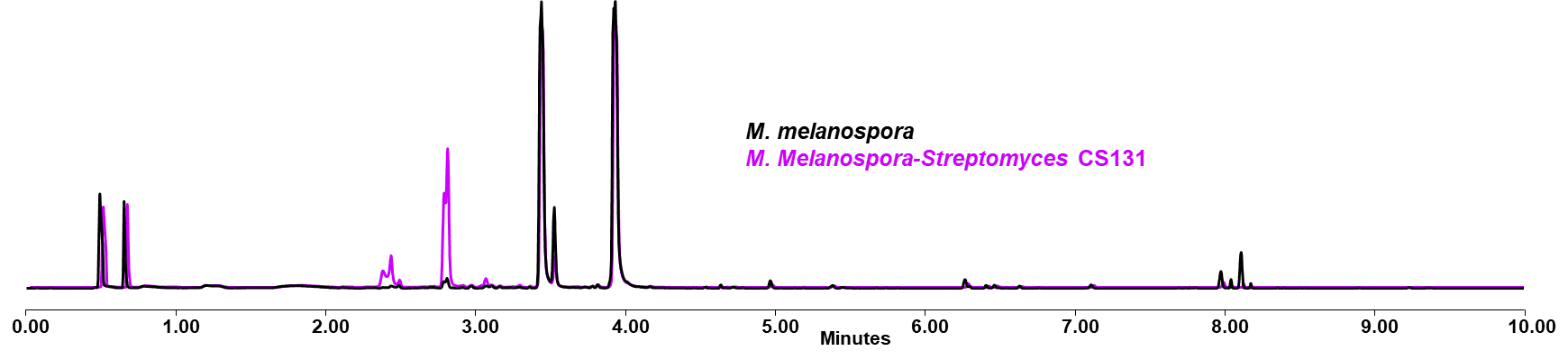


Additional Figure 4: Comparative UPLC profile of *M. melanospora* cultured on SFM against *Streptomyces* sp. CS131 and extracted with butanol. It can be appreciated the induction of the synthesis of various metabolites.
